# Microbial Colonization From the Fetus to Early Childhood—A Comprehensive Review

**DOI:** 10.3389/fcimb.2020.573735

**Published:** 2020-10-30

**Authors:** Viola Senn, Dirk Bassler, Rashikh Choudhury, Felix Scholkmann, Franziska Righini-Grunder, Raphael N. Vuille-dit-Bille, Tanja Restin

**Affiliations:** ^1^ Newborn Research Zurich, Department of Neonatology, University Hospital Zurich and University of Zurich, Zurich, Switzerland; ^2^ Division of Transplantation Surgery, Department of Surgery, University of Colorado Hospital, Aurora, CO, United States; ^3^ Division of Pediatric Gastroenterology, Hepatology and Nutrition, Children’s Hospital Lucerne, Lucerne, Switzerland; ^4^ Department of Pediatric Surgery, University Children’s Hospital of Basel, Basel, Switzerland; ^5^ Institute of Physiology, University of Zurich, Zurich, Switzerland

**Keywords:** microbiome, microbiota, fetus, newborn, infant

## Abstract

The development of the neonatal gastrointestinal tract microbiota remains a poorly understood process. The interplay between neonatal (gestational age, genetic background), maternal (mode of delivery, nutritional status) and environmental factors (antibiotic exposure, available nutrition) are thought to influence microbial colonization, however, the exact mechanisms are unclear. Derangements in this process likely contribute to various gastrointestinal diseases including necrotizing enterocolitis and inflammatory bowel disease. As such, enhanced understanding of microbiota development may hold the key to significantly reduce the burden of gastrointestinal disease in the pediatric population. The most debatable topics during microbial seeding and possible future treatment approaches will be highlighted in this review.

## Introduction

Humans are “holobionts”, which means that they host an assembly of their own human eukaryotic cells and all of the microorganisms living in/on them (Meyer-Abich, 1943; Margulis and Fester, 1991). It is well established that the human microbiota comprises a wide array of microorganisms including bacteria, archaea, fungi, and protozoa. The entirety of their corresponding genes are referred by the term “microbiome” as reviewed by Lynch et al. (Lynch and Pedersen, 2016). Because viruses are hosted in eukaryotic cells, bacteria or archaea, they are included under the umbrella of the microbiome as well (Virgin, 2014). Metagenomic data and new bioinformatic tools help to detect these hidden viral nucleotide sequences which may influence host phenotype (Angly et al., 2005; Virgin and Todd, 2011). The largest microbiota of the human body is found in the gastrointestinal tract (GIT) with about 10^13^–10^14^ microorganisms (Sender et al., 2016). As a nutritional inflow source, the GIT represents a fertile ground for microbial colonization. However, what types of microorganisms persist and in what quantity they do so, relies upon the methods by which microorganisms extract energy and provide commensal benefit to the GIT. The challenge of the host immune system is to both accept these commensal bacteria and defend against pathogens (Round and Mazmanian, 2009; Kim and Claud, 2019). Not only do resident microbiota extract energy for their survival, but they can also support the GIT in its function including pathogen defense (Freter, 1955; Abt and Pamer, 2014), strengthening the intestinal barrier function (Rakoff-Nahoum et al., 2004; Hayes et al., 2018) and promoting the immune development (O’Mahony et al., 2006; Round and Mazmanian, 2009). Additionally, the GIT microbiota helps to digests nutrients and improves gut motility (Abrams and Bishop, 1967; Dimidi et al., 2017) while supporting the synthesis of essential fatty acids (Høverstad and Midtvedt, 1986), amino acids (Jimenez et al., 2005), vitamins (Gustafsson et al., 1962) and hormones (Yano et al., 2015; Martin et al., 2019).

Existing literature suggests that children who are vaginally delivered at term without any instrumental assistance and are fed with maternal breast milk have the best chance to develop a healthy gastrointestinal microbiota which prevents dysbiosis (Levin et al., 2016; Martin et al., 2016). Dysbiosis refers to a phenomenon of microbiota “imbalance” or degeneracy in the microorganism make-up, which is thought to be associated with a wide range of metabolic/GIT diseases including obesity and metabolic syndrome (Turnbaugh et al., 2009), type 1 diabetes (Kostic et al., 2015), atopic conditions (Kalliomaki et al., 2001), inflammatory bowel disease (IBD) (Gevers et al., 2014), and necrotizing enterocolitis NEC (Fundora et al., 2020). As such, further understanding of dysbiosis is the first step to not only potentially prevent disease but also to offer hope for therapy.

This review summarizes the current evidence on the development of microbial colonization with a focus on factors which have been associated with dysbiosis including gestational age, mode of delivery, nutrition and antibiotic therapy.

## First Microbial Colonization

The initiation of microbial colonization remains a controversial topic in developmental biology. The theory of “sterile womb” purports that the healthy fetus develops in a sterile environment in utero (Th and Bettelheim, 1988) and that microbial colonization starts after birth with the exception of intra-uterine infections during pregnancy (Küstner, 1877; Tissier, 1900). This theory has been challenged when microbial components have been detected in the placenta (Aagaard et al., 2014; Collado et al., 2016) amniotic fluid (Collado et al., 2016), umbilical cord blood (Jimenez et al., 2005), meconium (Jimenez et al., 2008; Chu et al., 2017; Tapiainen et al., 2018), and fetal membranes (Steel et al., 2005), even after uncomplicated pregnancies with healthy term born newborns (Perez-Munoz et al., 2017; Stinson et al., 2019; Patton and Neu, 2020). These microbial particles have typically been detected by sensitive polymerase chain reaction (PCR) methods. 16S ribosomal RNA is derived from the prokaryotic ribosome and is used to attribute detected RNA to respective bacterial strains (Woese and Fox, 1977). In all these studies, PCR mean copy numbers were low. Lauder et al. reported 5.72 × 10^2^ gene copies for the maternal side and 1.2 × 10^2^ for the fetal side in samples which were extracted from 0.1–0.5 g placental tissue (Lauder et al., 2016). When Rackaityte et al. aimed to control for procedural and environmental contamination, they found only 23.5 operational taxonomic units (OTUs) with ≥5 sequence read counts per meconium sample. Additionally, they analyzed the intestines of early terminated pregnancies (20 ± 2.2 weeks of gestation) and detected bacterial structures on electron scans (Rackaityte et al., 2020). Several sources of microbial fetal encounters have been proposed including ascension from the genitourinary tract (Zervomanolakis et al., 2007) or passage *via* mucosal membranes such as the oral cavity or the GIT (Han et al., 2004) of pregnant women (Baker et al., 2018). The analysis of potential bacterial seeding in utero is heavily complicated by intraamniotic infection. This infection occurs with an incidence of 3.9% of all women giving birth (Woodd et al., 2019). It may initially appear clinically silent but increases one’s risk of preterm birth (Hillier et al., 1988). Joint diagnosis of histological chorioamnionitis and bacterial growth in amnion cultures was found to be as low as 27.7% (Queiros da Mota et al., 2013), that is why detection of placental microbial particles could also represent clinically inapparent infections.

Defenders of the sterile womb hypothesis attribute the detected microbial particles to contamination (Olomu et al., 2020), because there was no evidence of viability of the detected bacterial structures (Rackaityte et al., 2020). Lim et al. found neither microbial nor viral communities in their amnion fluid samples from healthy term pregnancies (Lim et al., 2018; Lim et al., 2019). Correspondingly, in healthy pregnancies, the attempts to cultivate viable bacteria from placental specimen has thus far failed (Kuperman et al., 2020). Additionally, recent placental analyses of more than 500 placental tissue specimen assessed both with 16S- and metagenomic analyses revealed that besides pathogens (*B streptococci*), no placental microbiome has been detectable (de Goffau et al., 2019).

An interesting theory, which may help to join the two conflicting observations is that particles derived from bacteria, fungi or viruses can be transported *via* the placenta to various fetal sites and thereby contribute to the priming of the fetal immune system (Wilcox and Jones, 2018). Microbial structures might then occasionally be detected depending on the sensitivity of the method.

It has been demonstrated that bacteria, as part of the maternal microbiota can be absorbed by immune cells (Rescigno et al., 2001). In theory, they could be transported *via* the blood stream or the lymphatic system into the placenta (Funkhouser and Bordenstein, 2013). Taking into account the immunological challenge at the maternal-fetal interface of the placenta (Ander et al., 2019), we suspect that there is also the possibility, that dead bacterial components are expressed on placental dendritic cells and may be taken over to the fetal side to prime the fetal immune system as suspected for allergens (Szepfalusi et al., 2000).

However, the number of microbial agents which have been described in placental tissue remains low. Tenericutes, Firmicutes (*Lactobacillus*), Actinobacteria (*Bifidobacterium*, *Propionibacterium*, *Rhodococcus*, *Streptomyces*), Bacteroidetes (*Bacteroides*, *Prevotella*), Proteobacteria (*E. coli*, *Neisseria*, *Enterobacteria*), and Fusobacteria have been found in the placenta of healthy newborns at term (Aagaard et al., 2014; Parnell et al., 2017). Most taxa presented as “placental microbiome” correspond to the taxa found in the maternal oral microbiome (Fardini et al., 2010; Aagaard et al., 2014). Furthermore, it has been suggested that oral infections such as periodontitis are linked to complicated pregnancies and may contribute to prematurity or neonatal sepsis as reviewed by Zi et al. (2014).

With regard to umbilical cord blood of healthy term newborns Actinobacteria (*Bifidobacterium*, *Propionibacterium*), Proteobacteria (*Escherichia*), Firmicutes (*Enterococcus*, *Staphylococcus*, *Streptococcus*), and Bacteroidetes (*Bacteroides*) have been detected (Jimenez et al., 2005). Similarities between the microbiota of meconium, placenta, and amnion fluid of healthy infants either suggest a certain prenatal microbial antigen transfer or a common source of contamination. However, Chu et al. describe different bacteria on the newborn skin, mouth and nose depending on the mode of delivery (Chu et al., 2017). Contrastingly, they found similar bacteria in the newborn meconium with many samples harboring highly abundant Escherichia and Klebsiella (abundance 14.3% and 6.4%, respectively), not detectable in any other body site, speculating for a different microbial source prior to birth. It is tempting to assume that immunological priming with microbial particles starts to shape the fetal immune system prior to birth (Chu et al., 2017). Despite numerous papers published on this field, the concerns of contamination remain unsolved. In the newest study investigating this issue, evidences were put forward for contamination as the origin of bacteria found in human placenta samples (Gschwind et al., 2020).

## Influence of Gestational Age

It has been well demonstrated that prematurity (birth before the completion of 37 weeks of pregnancy) may be triggered by intrauterine infections. Inflamed leaky or ruptured membranes facilitate the ascension of bacteria from the genitourinary tract (Hillier et al., 1995; Leitich et al., 2003). The gastrointestinal tract of premature infants is also known to have leaky barrier properties with a higher transepithelial and -mucosal permeability (Weaver et al., 1984a; Weaver et al., 1984b), impaired motility (Berseth, 1996), less active digestive enzymes (Demers-Mathieu et al., 2018) and lower absorption of nutrients (Neu and Koldovsky, 1996). Compared to their term counterparts, the immune system of premature infants displays fewer amounts of leukocytes, less proinflammatory cytokines, and less antibacterial peptides (Strunk et al., 2011; Melville and Moss, 2013).

Furthermore, preterm delivery is often linked to complicated pregnancies with a higher rate of caesarian sections and the use of prenatal antibiotics (Hill et al., 2017; Salvatore et al., 2019). The less mature newborns are, the longer time they have to spend in the neonatal intensive care unit (NICU) (Maier et al., 2018). They receive parenteral nutrition and/or enteral nutrition *via* nasogastric tubes (Viswanathan and Jadcherla, 2019). Additionally, these infants often need respiratory support (Shi et al., 2020). As a result of these well described, common consequences of prematurity, preterm infants often have a delayed development of their gastrointestinal bacterial microbiota, a lower bacterial load (Chernikova et al., 2018), fewer commensals, and obligate anaerobic bacteria and a higher number of pathogens such as Klebsiella pneumoniae and Clostridium difficile and facultative anaerobic bacteria (Dahl et al., 2018). The dominating taxa consist of Firmicutes (*Staphylococcus*, *Enterococcus*), Proteobacteria (*Enterobacteriaceae*, *Escherichia*, *Klebsiella*), Actinobacteria, and Bacteroidetes (*Bacteroides*) (Patel et al., 2016; Yuan et al., 2019). Their term counterparts are colonized with predominantly Actinobacteria (*Bifidobacterium*) (Penders et al., 2006) and Firmicutes (*Staphylococcus, Streptococcus*) (Palmer et al., 2007).

These microbial changes in the preterm infant may be associated with feeding intolerance (Ford et al., 2019; Salvatore et al., 2019), NEC (Baranowski and Claud, 2019), late-onset sepsis (LOS) (Stewart et al., 2017), and inferior long-term neurological outcomes (Niemarkt et al., 2019). Even if premature infants meet the «optimal microbial conditions» including vaginal delivery, nutrition with breast milk and no antibiotic therapy, the premature microbiome differs from the microbiome of term neonates (Leitich et al., 2003; Penders et al., 2006; Palmer et al., 2007). However, currently, there is no consensus concerning the exact time point when the microbiota of preterm and term infants align, with different studies reporting a time range between 4 months and 4 years (Dahl et al., 2018; Fouhy et al., 2019). A recent study of 5–11-year old children including 51 former preterm children (≤ 32 weeks of gestational age) could still find an inflammatory gut profile in the preterm group. The differences were attributed to a reduced gut phage richness (Jayasinghe et al., 2020).

## Influence of Mode of Delivery

The mode of delivery influences the newborn gastrointestinal microbiota. During vaginal delivery, the infant’s GIT is colonized with vaginal (Dominguez-Bello et al., 2010) and intestinal (Makino et al., 2011; Makino et al., 2013) bacteria from the mother. Consequently, the infant’s gastrointestinal microbiota is dominated by Actinobacteria (*Bifidobacterium*, *Atobium*) (Dominguez-Bello et al., 2010; Reyman et al., 2019; Shao et al., 2019; Yang et al., 2019), Firmicutes (*Lactobacillus*, *Megamonas*) (Dominguez-Bello et al., 2010; Kuang et al., 2016), Bacteroidetes (*Prevotella*, *Bacteroides, Parabacteroides*) (Dominguez-Bello et al., 2010; Wampach et al., 2018), Fusobacteria (*Sneathia*) (Dominguez-Bello et al., 2010), and Proteobacteria (*Shigella, Escherichia*) (Kuang et al., 2016; Wampach et al., 2018; Shao et al., 2019; Yang et al., 2019). Most of these bacteria produce short chain fatty acids (SCFA), which lower the luminal pH and thereby inhibit the colonization of pathogens (Nagpal and Yamashiro, 2018). It is suggested that compared to children delivered *via* caesarean section, children after vaginal delivery display a higher diversity (Akagawa et al., 2019), fewer *Staphylococci* (Wampach et al., 2018) and *C. difficile* in their microbiota (Adlerberth and Wold, 2009).

In contrast, birth by caesarean (C-) section is theorized to interrupt the microbial transmission from the mother to child that occurs during vaginal birth (Backhed et al., 2015; Hill et al., 2017). Consequently, the GIT will first be colonized with bacteria present on the maternal skin (Akagawa et al., 2019) or in the direct neonatal environment (Dominguez-Bello et al., 2010). The intestinal microbiota after C-section is characterized by delayed bacterial colonization (Martin et al., 2016) and reduced number/diversity (Azad et al., 2013), but an increased number of opportunistic pathogens related to the hospital environment (Toscano et al., 2017a; Shao et al., 2019). After C-section the neonatal microbiome of the GIT is dominated by Firmicutes (*Enterococcus*, *Staphylococcus*, *Streptococcus*, *Clostridium*, *Veillonella*) (Azad et al., 2013; Martin et al., 2016; Kuang et al., 2016; Shao et al., 2019), and Proteobacteria (*Klebsiella*, *Enterobacter*, *Haemophilus*) (Shao et al., 2019). In relation to vaginal delivery, there are fewer Bacteroides, Bifidobacteria and Lactobacillus as well as SCFA (Nagpal and Yamashiro, 2018), and there is a general imbalance of the gut microbiome (Hoang et al., 2020). These findings correlate with a higher intraluminal pH and lower inhibition of pathogens (Nagpal and Yamashiro, 2018). There is some debate whether or not contractions might help to increase the microbial transfer to the baby (Levin et al., 2016; Shao et al., 2019) and how heavily the data is biased by antibiotic use as recommended prior to skin incision (Gholitabar et al., 2011). Mothers after C-section often additionally display a lower breastfeeding rate (Hobbs et al., 2016). In summary, C-section might be a contributing factor in the development of dysbiosis. However, the treatment of newborns with gauze swabs full of vaginal microbiota «vaginal seeding» did not show any benefit on long term outcomes but harbors the risk of pathogen transfer such as herpes, *group B streptococci*, *Chlamydia trachomatis*, and *Neisseria gonorrhoeae* (Cunnington et al., 2016; Haahr et al., 2018).

## Influence of Nutrition

At the beginning of the 20^th^ century, people realized that increasing alarming mortality rates of newborns and infants were associated with reduced rates of breastfeeding (Wolf, 2003). In the modern era, it is well understood that maternal breastfeeding indeed significantly reduces newborn and infant mortality and morbidity and contributes to maternal health (Ip et al., 2007; Zhao et al., 2020). However, the reasons underlying this association are more complex than initial theories related to breast milk providing nutrients and reducing pathogen transfer. Consequently, the composition of breastmilk and its influence on microbial composition is a growing area of research (Hennet and Borsig, 2016), not only due to the billion-dollar market linked to formula.

Important drivers for microbial seeding in the infant gut are pre- and probiotics in human breast milk (Sanders et al., 2019). Prebiotics are food components, which are not digested by human enzymes, but can be metabolized by certain bacteria, promote their growth and contribute to the health benefits of the host (Gibson and Roberfroid, 1995; Gibson, 1998; Gibson et al., 2004). Human milk oligosaccharides (HMO) are prebiotics and the third most common component of breast milk after lactose and lipids (Urashima et al., 2012). The first HMO has been described in 1954 as “bifidus factor” (Gyorgy et al., 1954a; Gyorgy et al., 1954b; Gauhe et al., 1954). These HMOs are not digested by pancreatic enzymes, but reach the colon intact, where they promote the growth of Bifidobacteria, Bacteroides and Lactobacillus (Marcobal et al., 2010; Thongaram et al., 2017). The digestion of HMO produces SCFA (such as acetate, propionate and butyrate), which can be used as energy source and lower the luminal pH, which inhibits the colonization of pathogens (Yu et al., 2013; David et al., 2014). Interestingly, the amount of specific fucosyl-oligosaccharides secreted into the milk seems to depend on the genetic background of the mother and whether it is preterm- or term breastmilk (Gabrielli et al., 2011). In addition to lipids and carbohydrates, human breast milk harbors proteins (immunoglobulins, enzymes) as well as hormones, growth factors, nucleotides, leukocytes, cytokines, lysozyme, and lactoferrin as reviewed by Hennet and Borsig (2016). Breast-fed children have an intestinal microbiota mainly dominated by Bifidobacteria and Lactobacilli (Cooke et al., 2005; Backhed et al., 2015), Bacteroides (which can digest HMO) (Wang et al., 2015) as well as Staphylococcus (Stewart et al., 2018). In contrast, children drinking formula tend to have a higher bacterial diversity and in addition to *Bifidobacteriaceae*, *Clostridia*, *Enterococcus*, and *Enterobacteriaceae* are detected (Harmsen et al., 2000; Li et al., 2014; Timmerman et al., 2017). However, studies are inconsistent (Adlerberth and Wold, 2009), possibly because they use different analytical approaches and infant nutrition and environmental influences are difficult to control in a large infant cohort.

In addition to the benefits named above, nutrition based on human milk is associated with a higher feeding tolerance (Schanler et al., 1999), lower risk of NEC (Miller et al., 2018), obesity (Ma et al., 2020), and atopic diseases (Lodge et al., 2015). However, the studies analyzing newborn and infant nutrition differ considerably concerning the duration and the amount of human milk provided as well as whether children were exclusively breastfed or human milk was provided with the bottle.

Prebiotic supplements such as galacto- and fructo-oligosaccharides added to infant formula shall mimic the effect of natural HMOs (Lodge et al., 2015), but to date, have not been demonstrated to lead to a complete approximation of the newborn microbiota (Bakker-Zierikzee et al., 2005; Haarman and Knol, 2005). In fact, intestinal microbiota of formula fed infants had more potential pathogens (Benno et al., 1984; Bezirtzoglou et al., 2011) as compared to breastfed children; dominated by Firmicutes (*Staphylococcus*, *Streptococcus*, *Enterococcus*, *Lactobacillus, Clostridium*), Bacteroidetes (*Bacteroides*), Proteobacteria (*Enterobacteria*), and Actinobacteria (*Atopobium*) (Fallani et al., 2010; Stewart et al., 2018). Probiotics are substances that contain vital microorganisms, which confer health benefits on their host (Food and Argiculture Organization of the United Nations, 2002; Hill et al., 2014). These microorganisms may change the microbial composition (Frese et al., 2017). They are believed to improve the barrier function of the intestinal epithelia, modify the immune response and protect against pathogens due to competition for nutrients and colonization with potential pathogens (Servin, 2004; Athalye-Jape et al., 2018). It is hypothesized that the supplementation of probiotics reduces the time to complete enteral feeding (Samanta et al., 2009; Braga et al., 2011), the duration of hospitalization (Romeo et al., 2011), and morbidity and mortality (Barrington, 2011). The best evidence available in this regard involves the combination of Bifidobacteria and Lactobacilli (Chang et al., 2017). A recent Cochrane review on this topic found 24 trials including 5,529 infants, all assessing probiotic treatment of preterm infants <37 weeks gestational age or <2.500 g birth weight. This meta-analysis found a significantly reduced incidence of severe NEC (Bell stage II or more) relative risk (RR) of 0.43 (95% confidence interval (CI) 0.33–0.56) and a reduced mortality RR 0.65 (CI 0.52–0.81) (Hobbs et al., 2016). However, the timing and composition of the probiotic treatment seems to be very important, because both the combination of Lactobacillus rhamnosus and *Lactobacillus helveticus L* (Freedman et al., 2018) and the supplementation of Lactobacillus rhamnosus alone were not sufficient to improve the outcome in children with gastroenteritis (Schnadower et al., 2018). Experts therefore suggest a personalized approach (Zmora et al., 2018).

While cultivated bacteria from breast milk samples have been attributed to contamination (Dudgeon and Jewesbury, 1924; Wright, 1947), living non-pathogenic bacteria below a density of 10^5^ colony forming units/ml are now considered to be within normal range (Weaver et al., 2019), and potentially beneficial to newborn health (Toscano et al., 2017b). Several analyses have detected living bacteria such as Firmicutes (*Staphylococcus, Streptococcus, Peptostreptococcus, Enterococcus, Clostridia, Lactobacillus*), Actinobacteria (*Bifidobacterium*, *Corynebacterium*), Bacteroidetes (*Bacteroides*), and Proteobacteria (*Escherichia*, *Serratia*, *Pseudomonas*) in human breast milk. With the possibilities of large scale metagenomic analyses, it has now become possible to track the potential transfer of mobile genetic elements and antibiotic resistance genes *via* breast milk (Parnanen et al., 2018). Additionally, viable fungi have been cultured from breast milk samples recently at a density of ≥10^3^/ml with the highest rate of Malassezia, Candida, and Saccharomyces taxae (Boix-Amoros et al., 2017). Vertical viral transmission from the mother to her newborn is evident for cytomegalovirus (CMV) (Bardanzellu et al., 2019), human immunodeficiency virus (HIV) (Van de Perre et al., 2012), and human-T-lymphotrope virus (HTLV). However, in general, breast-feeding has been associated with a lower risk of viral infections (Arifeen et al., 2001; Bahl et al., 2005). This viral reduction parallels with the finding, that prophages are also more abundant in formula-fed infants (Liang et al., 2020). There is a currently contentious debate in this regard to where microbial particles found in the human breast milk derive from and why microbial loads are reported to be divers (Biagi et al., 2017). Potential sources include the adjacent skin and areola of the breast and bacteria, fungi or viral particles located in the newborn nasopharyngeal tract, deriving from their direct environment. Some bacterial transfer can also be explained by reverse flow from the larger milk ducts near the nipple to smaller collecting ducts and ductules (Ramsay et al., 2004). However, Urbaniak et al. also found bacterial particles in breast samples which were taken from non-lactating women during an operation (Urbaniak et al., 2016). Some studies have suggested the origin of the human breast milk microbiota is the maternal GIT, because the transfer of obligate anaerobes such as Bifidobacterium breve which has been detected in breast milk samples is not possible *via* skin contact (Jost et al., 2014). If this was the case, bacteria in the intestinal lumen of the maternal GIT could be taken up by immune cells, transferred *via* the blood and/or lymphatic system and then secreted into the breast milk. Such a connection between the maternal GIT microbiota and breast milk production could be the key to new treatment opportunities in lactating mothers.

In 2019, Togo et al. reported the successful cultivation of methanogenic archaea from breast milk samples (Togo et al., 2019). The amount of DNA was low with 2 log 10 copies DNA/ml, but they are still suggested to be important commensals due to their H2 reducing properties (Hansen et al., 2011; Bang and Schmitz, 2015). Another driving force for human microbial diversity which has been nearly overlooked for a long time is the human phageome (Manrique et al., 2016). This highly dynamic system creates a high predation pressure, may be introduced with microbes in breast milk and shapes the human microbiome (Rodriguez-Valera et al., 2009). There is growing evidence that the maternal intestinal microbiota is an important driver of breast milk composition, suggesting that new interventions to optimize infant health could already start prior or during pregnancy.

After the introduction of solid foods, the differences between breast milk and formula fed infants become smaller and the microbiota starts to resemble the adult microbiota (Backhed et al., 2015). Some authors suggest that not the introduction of solid food but rather the cessation of breast milk leads to the alignment with the adult microbiota (Backhed et al., 2015; Levin et al., 2016). Given the fact that the human diet seem so decisively to influence microbial properties, the microbiota is potentially ripe for therapeutic intervention (Ku et al., 2020), especially during the newborn period.

## Influence of Antibiotics

Unfortunately, globally the incidence of infections remains high in the newborn period (Fanos et al., 2007), and several diseases affecting newborns necessitate the administration of broad spectrum antibiotics (Isaacs, 2000; Gordon and Jeffery, 2005; Clark et al., 2006). Pre- as well as peri- and postnatal antibiotic therapy negatively influences the neonatal microbiota (Gibson et al., 2015; Tapiainen et al., 2019) and consequently the development of the infant’s immune system (Zeissig and Blumberg, 2014). Antibiotic therapy is associated with a lower number of commensal bacteria with delayed colonization with Bifidobacteria and Bacteroidetes (Coker et al., 2020; Eck et al., 2020) and a higher amount of potential pathogens (Aloisio et al., 2016). However, attempts to restore a healthy microbiota with probiotic treatment after antibiotic use even led to prolonged dysbiosis in healthy volunteers (Suez et al., 2018). In a cohort of infants aged 2–36 months, Yassour et al. observed that antibiotic treatment significantly reduced the strain diversity inducing a less stable microbiota. Moreover, bacteria increasingly acquired antibiotic resistance genes (Yassour et al., 2016). As such, the use of antibiotics increase the probability of fungal overgrowth (Kligman, 1952). Moreover, antibiotics may impact on long-term health outcomes such as modified nutrient absorption (Krajmalnik-Brown et al., 2012), lower vitamin production (LeBlanc et al., 2013), higher incidence of obesity (Dawson-Hahn and Rhee, 2019) and atopic diseases (Baron et al., 2020). The aim is to protect this delicate balance of bacterial and probably fungal interactions which support the healthy GIT-microbiota (Peleg et al., 2010). Thereby, ending antibiotic therapy expeditiously and narrowing antibiotic therapy should be encouraged for infants that do require antibiotics.

## Development of the Microbiota in Early Childhood

**Figure 1:**
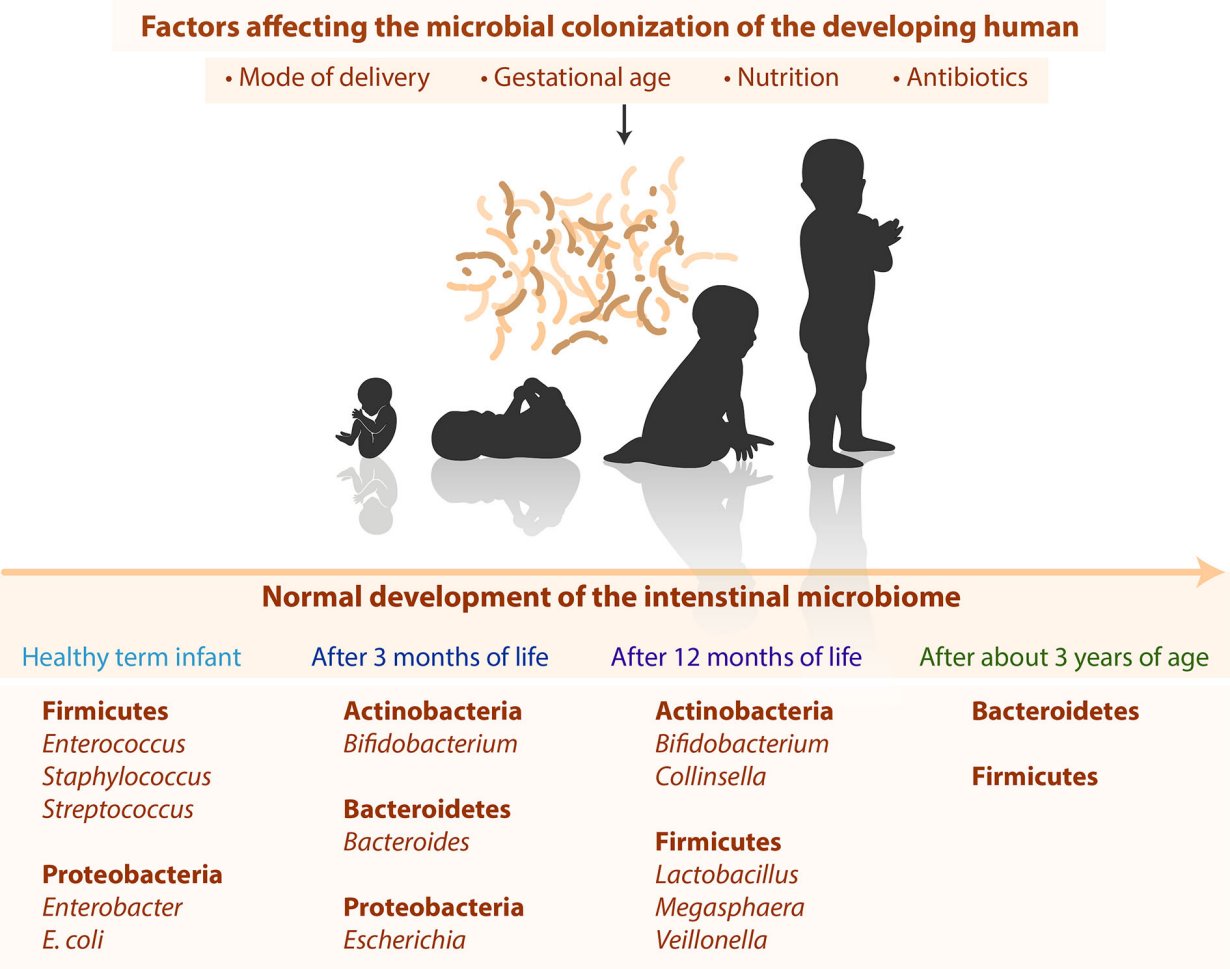
Summary of different factors and timely changes of the infant microbiome.

Despite huge individual differences (Eckburg et al., 2005; Ley et al., 2006; Turnbaugh et al., 2009) the development of the microbiota still follows typical timely changes as shown in **Figure 1**. Directly after birth, healthy term infants have a primarily aerobic GIT, which promotes the appearance of facultative anaerobe bacteria such as Firmicutes (*Enterococcus*, *Staphylococcus*, *Streptococcus*) and Proteobacteria (*Enterobacter, Escherichia coli*) (Palmer et al., 2007; Del Chierico et al., 2015). These bacteria reduce the oxygen content in the intestine and facilitate the occurrence of obligate anaerobic bacteria such as Actinobacteria (*Bifidobacterium*), Bacteroidetes (*Bacteroides*), and Firmicutes (*Clostridium, Lactobacillus, Ruminococcus*) (Koenig et al., 2011; Del Chierico et al., 2015). After three months of life, Actinobacteria (*Bifidobacterium*), Bacteroidetes (*Bacteroides*), and Proteobacteria (*Escherichia*) dominate the intestinal tract (Fallani et al., 2011; Hill et al., 2017). After 12 months of life, the infant’s GIT is dominated by Actinobacteria (*Bifidobacterium*, *Collinsella*) and Firmicutes (*Lactobacillus*, *Megasphaera*, *Veillonella*) (Penders et al., 2006). After 2–3 years of age, children display a higher diversity and lower inter-individual differences (Yatsunenko et al., 2012). The microbiota stabilizes after about 3 years and then resembles the adult microbiota in terms of diversity and complexity with high abundance of species from the firmicutes and bacteroidetes phyla (Arumugam et al., 2011).

## Discussion and Conclusion

This review demonstrates that there is still a large knowledge gap in regard to the microbial colonization of newborns. Neither the authors of the “sterile womb hypothesis” nor those defending the “in-utero colonization hypothesis” (Perez-Munoz et al., 2017) are able to completely explain the signaling mechanisms at the materno-fetal interface. Because the fetal intestinal immune system develops as early as 16 weeks of pregnancy (Stras et al., 2019) and fetal genetic particles have been found in the maternal blood (Lo et al., 1990; Lo et al., 1997), we assume that microbial particles derived from the mother are transported to the fetal side as well. This “microbial priming” (Ganal-Vonarburg et al., 2017) may help to prepare the offspring for microbial contact after birth. It could also be triggered *via* the transfer of bacterial, viral, archaeal or fungal components through the blood, the interstitium, or immune cells. Materno-fetal protein-transport and antigen-presentation has been previously described, for example *via* the placental Fc-receptors (Malek et al., 1998; Wilcox and Jones, 2018). Although exosomes become increasingly attentive (Czernek and Duchler, 2020), it is unclear, whether microbial particles might also be transferred *via* exosomes in healthy pregnancies.

The microbial seeding during the first days of life makes the newborn highly susceptible to microbial perturbations (Bokulich et al., 2016). The most important factors affecting microbial seeding are gestational age, mode of delivery, nutrition, and antibiotic therapy (Azad et al., 2016; Levin et al., 2016; Martin et al., 2016). Optimizing nutrition and medical treatment could potentially improve newborn growth, prevent NEC and support favorable long-term outcomes. However, the molecular mechanisms remain unclear. Additionally, most have used human feces as a surrogate to study the intestinal microbiota, although it is unknown to which extent the bacteria found in the feces represent the microbiota of the GIT and whether the luminal (transient) bacteria correlate with mucosal (resident) bacteria, which might differ depending on the gastrointestinal region (Sundin et al., 2020).

In summary, we are convinced that a deeper understanding of the development of the newborn and infant microbiota will help to discover further potentially modifying factors to improve long-term health and quality of life.

## Author Contribution

VS and TR conceptualized the draft and wrote the manuscript. FS provided the figure and revised the paper. DB, RC, FR-G, and RV critically reviewed and improved the manuscript. All authors finally approved this final version. All authors contributed to the article and approved the submitted version.

## Conflict of Interest

The authors declare that the research was conducted in the absence of any commercial or financial relationships that could be construed as a potential conflict of interest.
