# Corrigendum: Microbial Colonization From the Fetus to Early Childhood—A Comprehensive Review

**DOI:** 10.3389/fcimb.2021.715671

**Published:** 2021-06-30

**Authors:** Viola Senn, Dirk Bassler, Rashikh Choudhury, Felix Scholkmann, Franziska Righini-Grunder, Raphael N. Vuille-dit-Bille, Tanja Restin

**Affiliations:** ^1^ Newborn Research Zurich, Department of Neonatology, University Hospital Zurich and University of Zurich, Zurich, Switzerland; ^2^ Division of Transplantation Surgery, Department of Surgery, University of Colorado Hospital, Aurora, CO, United States; ^3^ Division of Pediatric Gastroenterology, Hepatology and Nutrition, Children’s Hospital Lucerne, Lucerne, Switzerland; ^4^ Department of Pediatric Surgery, University Children’s Hospital of Basel, Basel, Switzerland; ^5^ Institute of Physiology, University of Zurich, Zurich, Switzerland

**Keywords:** microbiome, microbiota, fetus, newborn, infant

## Incorrect Author Name

In the original article, an author name was incorrectly spelled as Raphael N. Vuille-dit-Bile. The correct spelling is Raphael N. Vuille-dit-Bille.

The authors apologize for this error and state that this does not change the scientific conclusions of the article in any way. The original article has been updated.

